# 20(S)-Protopanaxadiol-Induced Apoptosis in MCF-7 Breast Cancer Cell Line through the Inhibition of PI3K/AKT/mTOR Signaling Pathway

**DOI:** 10.3390/ijms19041053

**Published:** 2018-04-02

**Authors:** Hong Zhang, Hua-Li Xu, Yu-Chen Wang, Ze-Yuan Lu, Xiao-Feng Yu, Da-Yun Sui

**Affiliations:** 1 Department of Pharmacology, School of Pharmaceutical Sciences, Jilin University, Changchun 130021, China; zlzhoan@163.com (H.Z.); xhl@jlu.edu.cn (H.-L.X.); scarwyc@163.com (Y.-C.W.); zeyuanlu@jlu.edu.cn (Z.-Y.L.); 2 School of Materials Science and Engineering, South China University of Technology, Guangzhou 510640, China; 3 R&D Center, Guangzhou Ribobio Co., Ltd., Guangzhou 510663, China

**Keywords:** 20(S)-Protopanaxadiol, PI3K/AKT/mTOR, MCF-7, apoptosis

## Abstract

20(S)-Protopanaxadiol (PPD) is one of the major active metabolites of ginseng. It has been reported that 20(S)-PPD shows a broad spectrum of antitumor effects. Our research study aims were to investigate whether apoptosis of human breast cancer MCF-7 cells could be induced by 20(S)-PPD by targeting the Phosphatidylinositol 3-kinase/Protein kinase B/Mammalian target of rapamycin (PI3K/AKT/mTOR) signal pathway in vitro and in vivo. Cell cycle analysis was performed by Propidium Iodide (PI) staining. To overexpress and knock down the expression of mTOR, pcDNA3.1-mTOR and mTOR small interfering RNA (siRNA) transient transfection assays were used, respectively. Cell viability and apoptosis were evaluated by 3-(4,5-dimethylthiazol-2-yl)-2,5-diphenyltetrazolium bromide (MTT)-test and Annexin V /PI double-staining after transfection. The antitumor effect in vivo was determined by the nude mice xenograft assay. After 24 h of incubation, treatment with 20(S)-PPD could upregulate phosphorylated-Phosphatase and tensin homologue deleted on chromosome 10 (p-PTEN) expression and downregulate PI3K/AKT/mTOR-pathway protein expression. Moreover, G0/G1 cell cycle arrest in MCF-7 cells could be induced by 20(S)-PPD treatment at high concentrations. Furthermore, overexpression or knockdown of mTOR could inhibit or promote the apoptotic effects of 20(S)-PPD. In addition, tumor volumes were partially reduced by 20(S)-PPD at 100 mg/kg in a MCF-7 xenograft model. Immunohistochemical staining indicated a close relationship between the inhibition of tumor growth and the PI3K/AKT/mTOR signal pathway. PI3K/AKT/mTOR pathway-mediated apoptosis may be one of the potential mechanisms of 20(S)-PPD treatment.

## 1. Introduction

Globally, the most common cancer among women is breast cancer, which is also the second most common malignancy in morbidity. In the 2010s, there were 1.67 million patients of breast cancer (25% of all cancers in women) [1] and 520,000 incident cases of deaths (15% of all cancer deaths) worldwide [2]. Although most patients suffer from in situ breast cancer and can be treated surgically, the leading cause of death of this disease is distal recurrence, which is common. In the past few decades, the cytotoxic chemotherapy and targeted therapies have developed rapidly and the survival rate of patients has improved, but in the United States, still more than 40,000 patients die of breast cancer each year [3].

Human estrogen receptor α (ER) and epidermal growth factor receptor 2 (HER2) are closely related to the development of the incidence levels of breast cancer, which determine the molecular markers of breast cancer subtypes and the treatment of breast cancer programs. Therefore, a new target for treatment of breast cancer and the development of diagnostic markers could provide early and effective treatment. For breast cancer, common treatments include endocrine therapy, HER2 guide therapy, and cytotoxic therapy [4].

Recently, biological studies have shown that PI3K/AKT/mTOR signaling pathway, which is closely related to the activation of cancer cell growth, survival, and migration and drug resistance of targeted therapy [5,6,7,8], is abnormally activated in many cancers, including breast cancer. Moreover, some investigators suggest that breast cancer occurs mainly through two mechanisms: one is the amplification of HER2 or overexpression of the receptor tyrosine kinase (RTK) activation pathway; the second is that PI3K/AKT/mTOR pathway proteins undergo specific mutations [9,10]. Different breast cancer subtypes have different, unique PI3K/AKT/mTOR signaling pathway changes, which may result in different clinical manifestations, so a molecular characterization of each tumor subtype is required to develop a unique treatment therapy. Therefore, the identification and classification of PI3K/AKT/mTOR signaling pathway activation is closely related to the breast cancer subtypes [11], because it is susceptible to potential drug interventions, which selectively target tumors while leaving normal tissue alive [12,13].

**Figure 1:**
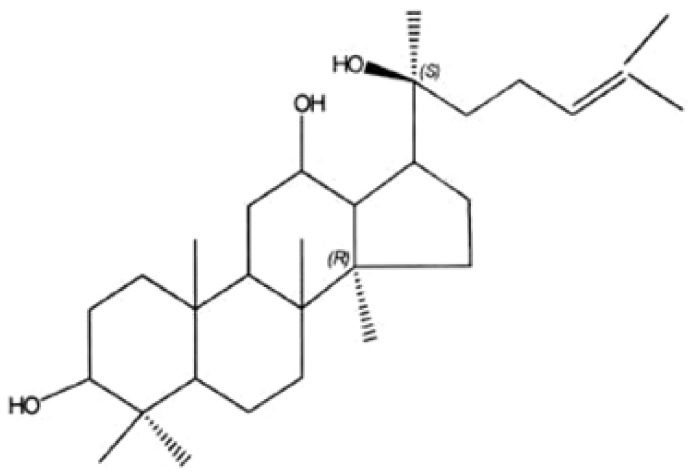
Chemical structure of 20(S)-protopanaxadiol (PPD). 20(S)-Protopanaxadiol (PPD) ((3β, 12β)-dammar-24-ene-3,12,20-triol; dammar-24-ene-3,12,20-triol, (3β, 12β)-; (3β, 5xi, 12β, 13α, 14β) -lanost-24-ene-3,12,20-triol, C_30_H_52_O_3_) is the final product of the ginseng diol group saponins, belonging to the tetracyclic triterpenoid compounds; its relative molecular mass is 460.70, its melting point is 197.5~198.5 °C, and the specific rotation is [α] D15 = 29.34° (C = 1.0, CHCl_3_).

20(S)-Protopanaxadiol (PPD), as one of the major active metabolites of ginseng, by human intestinal flora metabolism, is the final product of protopanaxadiol saponins (Figure 1) [14]. It has been reported that through caspase-dependent and caspase-independent pathways, 20(S)-PPD showed broad-spectrum antitumor effects in experimental animals and cultured cells [15,16,17,18]. At present, a Chinese medicine named the “Yijinsheng Capsule”, which has been developed from 20(S)-PPD, is currently in clinical phase 3 trials as a radiotherapy- and chemotherapy-assisting agent. It has been demonstrated that the AKT phosphorylation levels of A549, MCF-7, MGC803, SKOV3, Lncap, and Hep3B were significantly higher than in other cancer cell lines [19]. Our previous study indicated that in A549 cells, apoptosis could be induced by 20(S)-PPD treatment through the mitochondrial pathway and inhibition of AKT phosphorylation [20]. Moreover, further studies showed that in breast cancer MCF-7 cells, cell proliferation was inhibited and apoptosis was induced by 20(S)-PPD treatment [21]. In our present study, we investigated whether 20(S)-PPD may induce apoptosis in human breast cancer MCF-7 cells by targeting the PI3K/AKT/mTOR signaling pathway.

## 2. Results

### 2.1. 20(S)-PPD Inhibited the PI3K/AKT/mTOR Pathway and the Downstream Protein Expression in MCF-7 Cells

**Figure 2:**
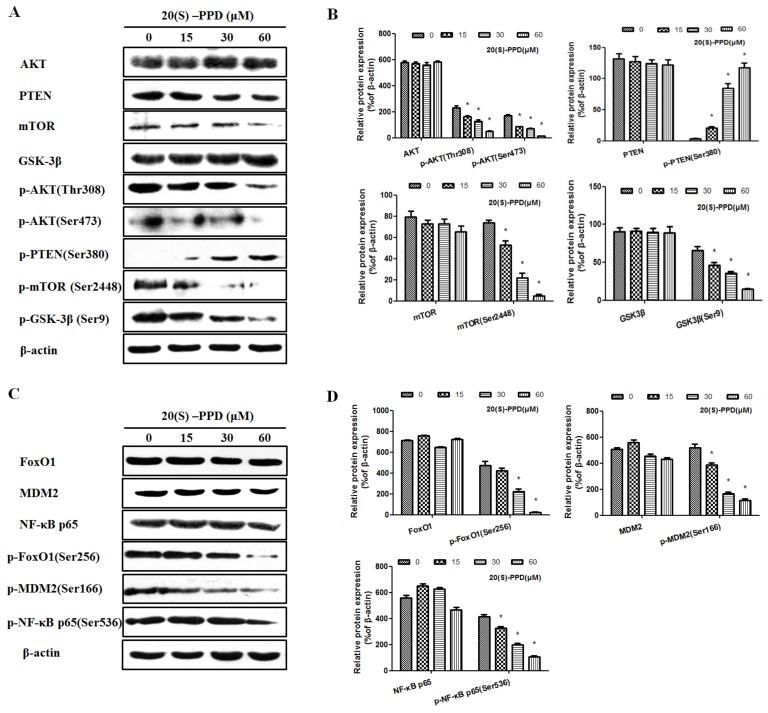
Effects of 20(S)-PPD on the PI3K/AKT/mTOR pathway and the downstream protein expression in MCF-7 cells. (**A**,**B**) Western blot analysis of the PI3K/AKT/mTOR signaling pathway-related proteins in MCF-7 cells after 20(S)-PPD (0, 15, 30, and 60 μM) administration for 24 h. (**C**,**D**) Expression of the downstream proteins Forkhead box protein O1 (FoxO1), (p-)FoxO1 (Ser256), Murine Double Mimute 2 (MDM2), p-MDM2 (Ser166), Nuclear Factor-κ-gene Binding (NF-κB) p65, and p-NF-κB p65 (Ser536) in MCF-7 cells after 20(S)-PPD treatment was detected by Western blot. All data were represented as mean ± S.D. * *p* < 0.05 compared to 0 μM.

We investigated the in vitro effect of 20(S)-PPD on the PI3K/AKT/mTOR signaling pathway by incubating MCF-7 cells with different concentrations of 20(S)-PPD (0, 15, 30, and 60 μM) for 24 h. As shown in Figure 2, Western blot analysis demonstrated that with the increasing of the concentration of 20(S)-PPD, the level of p-PTEN was increased, while the expression of p-AKT (Thr308), p-AKT (Ser473), p-mTOR (Ser2448), p-FoxO1 (Ser256), p-MDM2 (Ser166), p-NF-κB p65 (Ser536), and p-GSK-3β (Ser9) were markedly decreased. No significant changes were observed on the expression of AKT, PTEN, GSK-3β, mTOR, FoxO1, MDM2, and NF-κB p65 after 20(S)-PPD treatment.

### 2.2. 20(S)-PPD-Induced Cell Cycle Arrest of G1/G0 Phase in MCF-7 Cells

**Figure 3:**
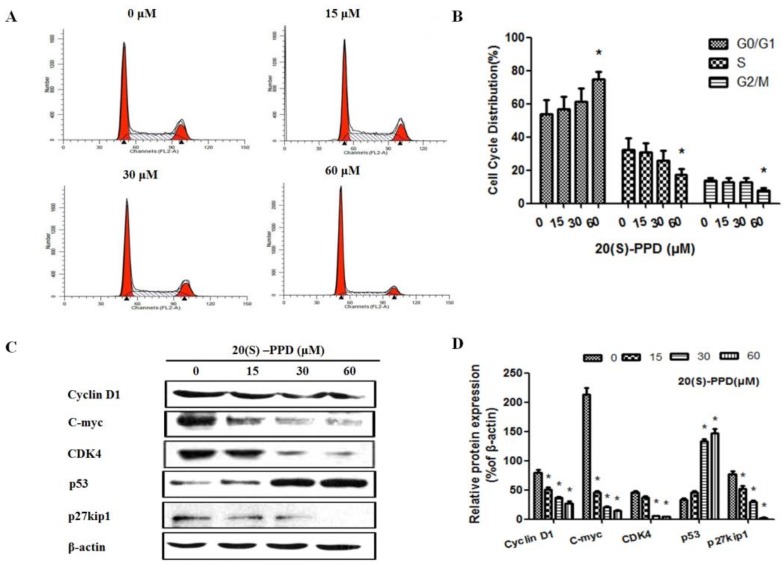
Effects of 20(S)-PPD on cell cycle arrest and the arrest-related proteins in MCF-7 cells. (**A**,**B**) Flow cytometry was used to detect cell cycle distribution. After 24 h treatment with 20(S)-PPD (0, 15, 30, and 60 μM), a propidium iodide (PI) staining assay was performed on MCF-7 cells. (**C**,**D**) In MCF-7 cells treated with 20(S)-PPD, the expression of cell cycle arrest-related proteins p53, p27^kip1^, c-myc, CDK 4, and cyclin D1 was detected by Western blot. G0 phase is a resting phase where the cell has left the cycle and has stopped dividing. G1 Phase is the first phase within interphase, from the end of the previous M phase until the beginning of DNA synthesis. S phase starts when DNA synthesis commences, when it is complete, all of the chromosomes have been replicated. G2 phase occurs after DNA replication and is a period of protein synthesis and rapid cell growth to prepare the cell for mitosis. M phase is called chromosome separation phase. All data were represented as mean ± S.D. * *p* < 0.05 compared to 0 μM.

In order to examine whether cell cycle arrest was related to 20(S)-PPD-inhibited MCF-7 cell growth, PI staining was used to perform cell cycle analysis. As shown in Figure 3A, G0/G1 cell cycle arrest could be induced by treatment with 20(S)-PPD in MCF-7 cells at 60 μM (*p* < 0.05), and the percentage of cells in G0/G1 phase were 53.89 ± 8.55%, 56.62 ± 7.62%, 61.31 ± 8.12%, and 74.61 ± 4.67%, respectively (Figure 3B). To investigate the underlying mechanism of G0/G1 cell arrest induced by 20(S)-PPD, Western blot was used to analyze cell cycle regulatory proteins. After 20(S)-PPD treatment, the level of p53 was markedly elevated and the expression of p27kip1, c-myc, CDK 4, and cyclin D1 were dramatically decreased by varying degrees at high doses (30–60 μM) (Figure 3C,D). These data suggest that 20(S)-PPD exhibited cell viability inhibition of MCF-7 cells via inducing G0/G1 phase cell arrest.

### 2.3. 20(S)-PPD-Induced Apoptosis Was Reversed by Transfection with mTOR Plasmid

**Figure 4:**
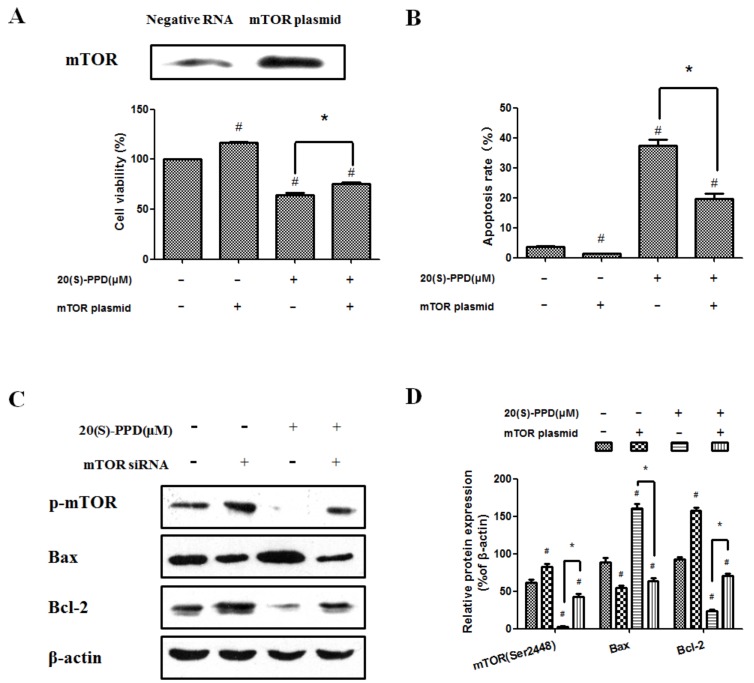
20(S)-PPD-induced apoptosis was reversed by transfection with mTOR plasmid. (**A**) Expression of mTOR after transfection of mTOR plasmid was viewed by Western blot (upper line). After 20(S)-PPD (30 μM) treatment in MCF-7 cells for 24 h, the MTT assay was used to determine the cell viability (lower line). (**B**) Flow cytometry was used to measure the apoptosis rate after 20(S)-PPD (30 μM) treatment for 24 h. (**C**,**D**) After 20(S)-PPD (30 μM) treatment of MCF-7 cells for 24 h, Western blot was used to determine the expression of Bax, Bcl-2 and p-mTOR. All data presented were represented as mean ± S.D. ^#^ *p* < 0.05 compared to control group, * *p* < 0.05 compared to 20(S)-PPD (30 μM) group.

To determine whether the PI3K/AKT/mTOR signaling pathway played a leading role of 20(S)-PPD-induced MCF-7 cell apoptosis, mTOR plasmid was transiently transfected into the cells that were subsequently incubated with 20(S)-PPD (30 μM) for 24 h. After transfection with mTOR plasmid, the expression of mTOR was upregulated significantly, as seen by Western blot analysis, and cell viability was also increased compared with treatment with 20(S)-PPD (30 μM) only (Figure 4A). As shown in Figure 4B, transfection with mTOR plasmid could weaken the effect of 20(S)-PPD-induced apoptosis. In addition, Western blot analysis indicated that the protein expression of Bax, Bcl-2, and p-mTOR (Ser2448) were regulated by 20(S)-PPD, and these effects mediated by 20(S)-PPD were partially reversed by transfection with mTOR plasmid (Figure 4C,D).

### 2.4. 20(S)-PPD-Induced Apoptosis Was Promoted by Knockdown of mTOR with siRNA

**Figure 5:**
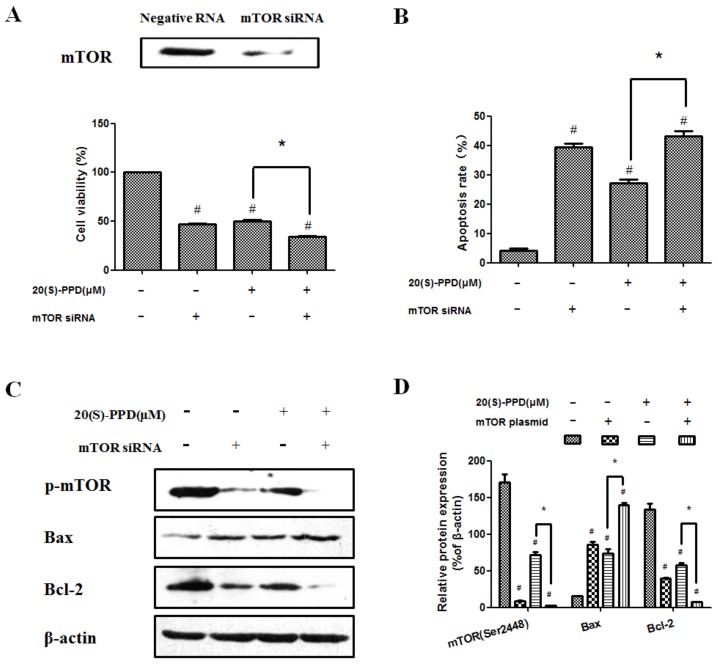
20(S)-PPD-induced apoptosis was promoted by knockdown of mTOR with siRNA. (**A**) Western blot was used to detect mTOR expression after siRNA transfection (upper line). After 20(S)-PPD (30 μM) treatment in MCF-7 cells for 24 h, the MTT assay was used to determine the cell viability (lower line). (**B**) Flow cytometry was used to measure the apoptosis rate after 20(S)-PPD (30 μM) treatment for 24 h. (**C**,**D**) After 20(S)-PPD (30 μM) treatment of MCF-7 cells for 24 h, Western blot was used to determine the expression of Bax, Bcl-2, and p-mTOR. All data presented were represented as mean ± S.D. ^#^ *p* < 0.05 compared to the control group; * *p* < 0.05 compared to the 20(S)-PPD (30 μM) group.

To further examine whether 20(S)-PPD-induced apoptosis involves the PI3K/AKT/mTOR signaling pathway, MCF-7 cells were transiently transfected with mTOR siRNA. The expression of mTOR was downregulated significantly after mTOR siRNA transfection and cell viability was decreased compared with treatment with 20(S)-PPD (30 μM) only (Figure 5A). As shown in Figure 5B, the combination of treatment with 20(S)-PPD and knockdown of mTOR with siRNA could further enhance the apoptotic effect induced by 20(S)-PPD (30 μM) only. Moreover, knockdown of mTOR with siRNA could promote 20(S)-PPD-induced apoptosis by regulating the protein expression of Bax, Bcl-2, and p-mTOR (Ser2448) (Figure 5C,D).

### 2.5. 20(S)-PPD Inhibited the Growth of MCF-7 Breast Cancer Cells in a Nude Mice Xenograft Assay

**Figure 6:**
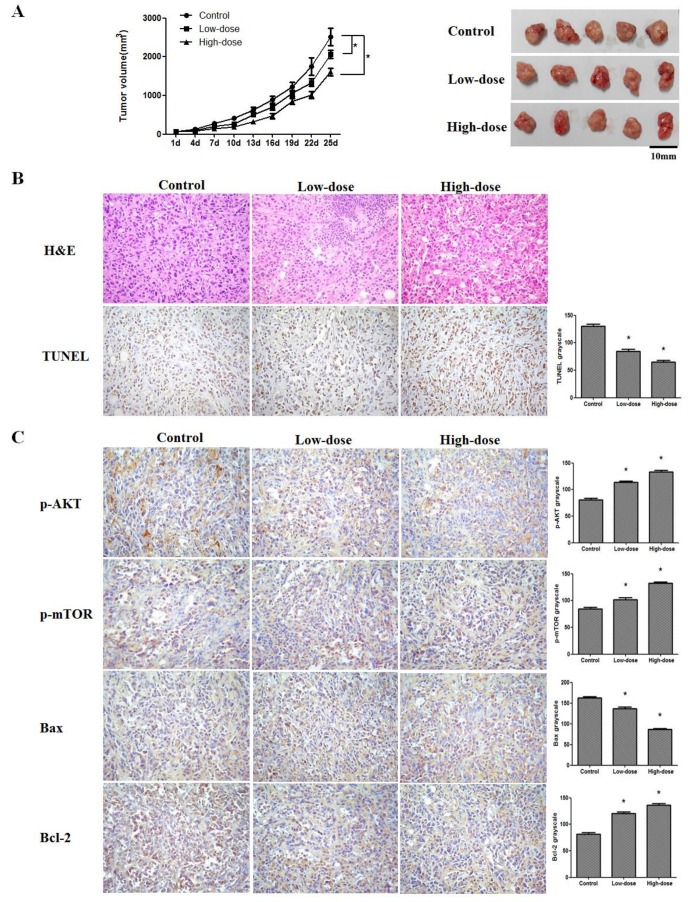
Effects of 20(S)-PPD on the nude mice xenograft of MCF-7 cells in vivo. (**A**) Tumor growth curves of the control and 20(S)-PPD (50,100 mg/kg) treatment groups were drawn by measuring and calculating tumor volume once every three days. (**B**) Excised human breast tumor images from the experimental group. (**C**) H&E staining, TUNEL assay, and immunohistochemistry were used to detect DNA damage, apoptosis, and p-AKT, p-mTOR, Bax, and Bcl-2 expression in tumor xenograft tissues (100×), respectively, and these data were represented by grayscale: the darker the grayscale is, the lower the protein expression. All data presented were represented as mean ± S.D. * *p* < 0.05 compared to the control group.

To evaluate whether 20(S)-PPD exhibited tumor growth inhibition in vivo, female BALB/c nude mice were injected subcutaneously with 0.2 mL human breast cancer MCF-7 cell suspension (1 × 10^7^ cells/mL and Matrigel basement membrane matrix at a 1:1 ratio) in the right flank. After being administrated orally for 25 days (d), 20(S)-PPD at high dosage (100 mg/kg) could partially suppress the tumor growth of MCF-7 cells (Figure 6A). In the control group, the average tumor size at 25 d was 2514.9 ± 221.7 mm^3^, whereas in the low-dose (50 mg/kg) and high-dose (100 mg/kg) treated groups, the average tumor volume was 2065.1 ± 105.2 and 1609.1 ± 96.2 mm^3^, respectively. In addition, we did not observe significant impairment of hepatotoxicity, nephrotoxicity, cardiotoxicity, and other immune organ toxicity in the mice after treatment with 20(S)-PPD (data not shown). Furthermore, the MCF-7 tumor from the control and 20(S)-PPD-treated mice were harvested, and immunohistochemistry analysis was used to assess the PI3K/AKT/mTOR pathway and apoptosis. As shown in Figure 6B, a large number of hemorrhage- and necrosis-affected cells appeared after 20(S)-PPD treatment, detected by H&E staining, and apoptotic cells in tumor tissues were detected by Terminal deoxynucleotidyl transferase dUTP nick end labeling (TUNEL) assay. These data showed that DNA damage of tumor tissues was induced by 20(S)-PPD. In addition, immunohistochemistry analysis displayed that the phospho-AKT, phospho-mTOR, and Bcl-2 levels were significantly reduced and that the Bax level was increased (Figure 6C).

## 3. Discussion

One of the most common female malignancies is breast cancer, which shows high incidence and high mortality. According to statistics, the incidence of breast cancer accounts for 7–10% of various malignant tumors [22,23]. It has been reported that PI3K/AKT/mTOR pathway proteins are affected by specific mutations in breast cancer. Moreover, 20(S)-PPD could inhibit breast cancer MCF-7 cell proliferation and induce cell apoptosis via a caspase-mediated apoptosis pathway [21]. Thus, we make a hypothesis that the human breast cancer MCF-7 cell apoptosis induced by 20(S)-PPD may be related to targeting of the PI3K/AKT/mTOR signal pathway.

The PI3K/AKT/mTOR signaling pathway, which is frequently abnormal in many tumor cells, is becoming an important target in cancer therapy. AKT activation is mediated by PIP3, which is the product of PI3K and is also an important substrate of PTEN. In addition, PIP3 was dephosphorylated by PTEN, which reduces the expression of the PI3K/AKT pathway, leading to cell apoptosis [24]. These data apparently suggest that 20(S)-PPD at high concentrations (30–60 μM) can inhibit the PI3K/AKT/mTOR pathway and phosphorylation of its downstream targets (Figure 2). We also determined whether 20(S)-PPD inhibited tumor growth in vivo. 20(S)-PPD (100 mg/kg) could partially suppress the tumor growth of MCF-7 cells after 25 d of treatment, and the tumor growth inhibition (TGI) was about 40%. Furthermore, a TUNEL assay detected the apoptotic cells of tumor tissues, and the data showed that DNA damage of tumor tissues was induced by 20(S)-PPD. In addition, immunohistochemistry data show that the Bax expression was elevated and that phospho-AKT, phospho-mTOR, and Bcl-2 expression were reduced significantly (Figure 6). These data suggest that the inhibition of tumor growth may relate to the apoptosis induced by 20(S)-PPD through the PI3K/AKT/mTOR pathway.

Induction of cell cycle arrest in cancer cells has become one of the main indicators of anticancer effects. Regulation of the cell-cycle machinery may be altered by many anticancer agents: this may lead to cell cycle arrest during any phase, and eventually result in inhibition of the growth and proliferation of cancerous cells, especially in breast cancer [25,26]. It is reported that G0/G1 arrest in cancer cells is induced by inhibition of the PI3K/AKT/mTOR pathway [27]. Accordingly, PI staining was used to carry out cell-cycle analysis of the MCF-7 cells. In our study, G0/G1 cell cycle arrest in MCF-7 cells could be induced by 20(S)-PPD at high doses (>30 μM), which was consistent with previous research studies. A number of well-known downstream targets translationally regulated by PI3K/AKT/mTOR, including the cell cycle-related proteins CDK4, c-Myc, cyclin D1, and p27kip1, were suppressed concomitantly by 20(S)-PPD, but p53 was markedly elevated by 20(S)-PPD (Figure 3). Cyclin D1, CDK4, c-Myc, and p27kip1 contribute to tumor growth by promoting cell cycle progression [28,29,30]. Therefore, 20(S)-PPD at high doses inhibits MCF-7 cell proliferation and induces apoptosis likely by a mechanism that involves a strong G0/G1 arrest and the regulation of cell cycle-related proteins.

To investigate whether 20(S)-PPD treatment could regulate apoptosis by targeting the PI3K/AKT/mTOR signaling pathway, MCF-7 cells were transiently transfected with mTOR plasmid or mTOR siRNA, respectively. Transfected mTOR plasmid in MCF-7 cells could significantly upregulate the expression of mTOR, increased the cell viability, and partially reversed (did not bring back apoptosis to normal levels) the effect of 20(S)-PPD-induced apoptosis (Figure 4). These data suggest that the PI3K/AKT/mTOR signaling pathway is not the only mechanism of 20(S)-PPD-mediated apoptosis and there may be also other molecular pathways. While transiently transfected with mTOR, siRNA could significantly downregulate the expression of mTOR and decrease cell viability. Moreover, the apoptotic effect of 20(S)-PPD could be promoted by knockdown of mTOR with siRNA through regulating the protein expression of Bax, Bcl-2, and p-mTOR (Ser2448) (Figure 5). These data demonstrate that the PI3K/AKT/mTOR signaling pathway is one of the potential mechanisms whereby 20(S)-PPD induces MCF-7 cell apoptosis.

## 4. Materials and Methods

### 4.1. Reagents and Antibodies

Hainan Asia Pharmaceutical Co. Led., (Haikou, China) provided experimental use 20(S)-protopanaxadiol (PPD) and its purity was >95% detected by HPLC. Antibodies against AKT, phospho-AKT (Thr308/Ser473), c-myc, Cyclin D1, CDK4, FoxO1, phospho-FoxO1 (Ser256), GSK-3β, phospho-GSK-3β (Ser9), mTOR, phospho-mTOR (Ser2448), MDM2, phospho-MDM2 (Ser166), NF-κB p65, phospho-NF-κB p65 (Ser536), PTEN, phospho-PTEN (Ser380), p53, p27kip1, pcDNA3.1-mTOR, mTOR siRNA, and negative control RNA were obtained from Cell Signal Technology (Boston, MA, USA). β-actin antibody was purchased from Tianjin Jingmai (Tianjin, China). Antibodies against phospho-AKT (Ser473), phospho-mTOR (Ser2448), Bcl-2, and Bax used for immunohistochemistry were obtained from Beijing Biosynthesis Biotechnology Co., Ltd. (Beijing, China). BCA protein assay reagent kit was purchased from Beyotime Institute of Biotechnology (Shanghai, China). In Situ Cell Death Detection Kit, Peroxidase (POD) was purchased from Roche (Basel, Switzerland). Annexin V apoptosis detection kit was purchased from Tianjin Sungene Biotech Co. Ltd. (Tianjin, China). Dolichos bifows agglutinin (DBA) and Streptavidin-peroxidase (SP) kits were obtained from Fuzhou Maixin Biotechnology Co., Ltd. (Fuzhou, Fujian, China). MTT, PI, and all other reagents were obtained from Sigma-Adrich Co. (St. Louis, MO, USA).

### 4.2. Cell Culture and Cell Viability Assay

Human breast cancer MCF-7 cells were purchased from Shanghai Institute of Cell Biology, Chinese Academic of Science (Shanghai, China). RPMI-1640 medium (Hyclone, Marlborough, MA, USA) supplemented with 10% heat-inactivated (56 °C, 30 min) fetal calf serum (FBS, GIBCO, Waltham, MA, USA) was used to maintain MCF-7 cells at standard conditions (37 °C, 95:5% mixed humidified air and CO_2_). 20(S)-PPD was dissolved with DMSO and added to the culture media to the final concentrations, and the final DMSO concentration was less than 0.1%.

MTT assay was used to detect cell viability as described previously [20]. Briefly, the MCF-7 cells after transient transfection or not were seeded into 96-well plates. After being cultured at standard conditions for 24 h, MCF-7 cells were incubated with or without 20(S)-PPD. After 20 h, 10 μL of MTT (Sigma, 5 mg/mL in PBS, St. Louis, MO, USA) solution was added to each well and then incubated for another 4 h. Then, the supernatant was discarded and 100 μL of DMSO was added to each well, shaking the plates for 10 min. The microplate reader (SpectraMax Plus384, Molecular Devices, San Jose, CA, USA) was used to detect the absorbance at 570 nm.

### 4.3. Apoptosis Assessment

The apoptosis rate of MCF-7 cells was quantified by Annexin V/PI staining. As previously described, after transient transfection or not, cells were treated with or without 20(S)-PPD (30 μM). After 24 h of culturing in standard conditions, we harvested the cells and washed them twice with PBS. After centrifugation, MCF-7 cells were resuspended with 1× binding buffer containing PI (1 μg/mL) and Annexin V (0.05 μg/mL). After a 15-min incubation in the dark at room temperature, flow cytometry was performed to analyze the samples.

### 4.4. Western Blot Analysis

The expression of specific proteins was detected by Western blot analysis as described previously [21]. After 20(S)-PPD treatment at different concentrations for 24 h, we collected the cells and added RIPA buffer to lyse on ice for 30 min. According to the BCA protein assay kit protocol, the protein concentration was determined. 12% polyacrylamide-SDS gel was used to separate the total cell extracts (20 μg). After electrophoresis, the gel was transferred onto a PVDF (Poly vinylidense difluoride) membrane; the membrane was blocked with 5% (*w*/*v*) nonfat milk for 1 h and then overnight at 4 °C with the primary antibodies described previously. HRP (Horseradish peroxidase)-conjugated secondary antibody was used to detect primary antibody binding and ECL (Enhanced chemiluminescence) was used to visualize it.

### 4.5. Cell Cycle Analysis

PI single staining was used to perform the cell cycle assay. At first, MCF-7 cells were incubated with 20(S)-PPD at different concentrations for 24 h. Then, the cells were trypsinized and ice-cold 70% absolute ethanol was used to resuspend and store them at −20 °C overnight. Cell cycle assay buffer was prepared as described previously (0.1 mg/mL RNase A and 50 mg/mL propidium iodide (PI) into PBS (pH 7.4)) and added to the cells at room temperature for 30 min, avoiding light. Finally, flow cytometry was used to determine the percentage of cells in different phases of the cell cycle.

### 4.6. Transfection Assay

Overexpression and knockdown of expression of mTOR were achieved by transient transfection with pcDNA3.1-mTOR and mTOR siRNA, respectively. Following the manufacturer’s instructions, MCF-7 cells were transfected with negative control RNA or pcDNA3.1-mTOR/mTOR siRNA at a concentration of 50 nM using Lipofectamine2000 (Invitrogen, Carlsbad, CA, USA). After 24 h of transfection, MCF-7 cells were incubated with or without 20(S)-PPD (30 μM) for another 24 h and then harvested to detect cell viability, apoptosis rate, and protein expression.

### 4.7. In Vivo MCF-7 Cell Xenograft Antitumor Research

The study was authorized by the Institutional Animal Ethical Committee of Jilin University and performed in an SPF (Specefic pathogen free) class laboratory. Female BALB/c nude mice were obtained from Beijing Vital River Laboratory Animal Technology Co., Ltd. (Beijing, China). Six-week-old mice were used for MCF-7 xenografted mice experiments. MCF-7 cells were adjusted to a concentration of 1.0 × 10^7^ cells suspended in 100 µL serum-free RPMI-1640. The cell suspensions with 100 µL Matrigel (Becton Dickinson, Bedford, MA, USA) were then injected subcutaneously into the right flanks of BALB/c nude mice. Tumor development was checked by sequential caliper measurements of length (L) and width (W). Tumor volume was calculated as volume  =  L × W2 × π/6. When the average volume of tumors reached 100–150 mm^3^, the mice were grouped randomly according to the tumor volume and administered orally with vehicle or 20(S)-PPD (50,100 mg/kg) every day. Developed tumors were resected 25 days after xenografts. Using general anesthesia (sevoflurane, Valisi Chemical Co., Ltd., Shanghai, China), tumor tissue was excised. Resected tissues were cut into 5-mm^3^ specimens and fixed in 10% neutral-buffered formalin for histological analysis.

### 4.8. Immunohistochemistry and H&E Staining

Tumor tissues of xenografted mice were resected as described above. The tissues were fixed in 10% neutral-buffered formalin. After being dehydrated and coated with wax, tissue sections were sliced to a thickness of 4 μm and then they were dyed using either the primary antibody for immunostaining or hematoxylin and erosin (H&E). A Nikon TS100 microscope (Nikon Corporation, Tokyo, Japan) was used to capture the staining results.

### 4.9. TUNEL Staining

Tumor tissues of xenografted mice were resected as described above. The tissues were fixed in 10% neutral-buffered formalin. After being dewaxed and hydrated, the tumors underwent TUNEL staining following the manufacturer’s instructions (Roche, Basel, Switzerland). We randomly chose four fields to analyze the results and defined the apoptotic index as follows: apoptotic index (%) = 100 × apoptotic cells/total tumor cells.

### 4.10. Statistical Analysis

All data were represented as mean ± standard deviations for three independent experiments. One-way analysis of variance (ANOVA) or Student’s test were used to perform statistical differences. The statistically significant standard was *p* values of <0.05.

## 5. Conclusions

The growth of breast cancer MCF-7 cells can be inhibited by treatment with 20(S)-PPD at high doses, in vitro and in vivo. One of the potential mechanisms may be attributed to PI3K/AKT/mTOR pathway-mediated apoptosis. As an aglycone saponin ginsenoside, 20(S)-PPD appears to be a promising orally active anticancer drug and may be developed into a promising adjuvant agent for radiotherapy and chemotherapy against breast cancer.
